# Dominant male song performance reflects current immune state in a cooperatively breeding songbird

**DOI:** 10.1002/ece3.1938

**Published:** 2016-01-20

**Authors:** Jenny E. York, Andrew N. Radford, Ton G. Groothuis, Andrew J. Young

**Affiliations:** ^1^ Centre for Ecology and ConservationCollege of Life and Environmental SciencesUniversity of Exeter, Penryn Campus, Treliever Road, PenrynCornwallTR10 9EZUK; ^2^ School of Biological SciencesUniversity of Bristol24 Tyndall AvenueBristolBS8 1TQUK; ^3^ Department of ZoologyUniversity of CambridgeDowning StreetCambridgeCB2 3EJUK; ^4^ Groningen Institute for Evolutionary Life SciencesUniversity of GroningenNijenborgh 79747AG GroningenThe Netherlands

**Keywords:** Behavioral plasticity, cooperative breeder, dominance, handicap hypothesis, resource‐allocation trade‐offs, sociality, state‐dependent signaling

## Abstract

Conspicuous displays are thought to have evolved as signals of individual “quality”, though precisely what they encode remains a focus of debate. While high quality signals may be produced by high quality individuals due to “good genes” or favourable early‐life conditions, whether current immune state also impacts signalling performance remains poorly understood, particularly in social species. Here, we experimentally demonstrate that male song performance is impaired by immune system activation in the cooperatively breeding white‐browed sparrow weaver (*Plocepasser mahali*). We experimentally activated the immune system of free‐living dominant males via subcutaneous injection of phytohemagglutinin (PHA) and contrasted its effects with those of a control (phosphate buffered saline) injection. PHA‐challenged males showed significant reductions in both the duration and the rate of their song performance, relative to controls, and this could not be readily attributed to effects of the challenge on body mass, as no such effects were detected. Furthermore, male song performance prior to immune‐challenge predicted the scale of the inflammatory response to the challenge. Our findings suggest that song performance characteristics are impacted by current immune state. This link between current state and signal performance might therefore contribute to enforcing the honesty of signal performance characteristics. Impacts of current state on signaling may be of particular importance in social species, where subordinates may benefit from an ability to identify and subsequently challenge same‐sex dominants in a weakened state.

## Introduction

Conspicuous signals are widely considered to encode information about individual quality. A number of mechanisms may generate relationships between aspects of an individual's quality and their signaling characteristics, such as the impacts of heritable genetic variation, historical health or early‐life developmental stress (Hamilton and Zuk 1982; Spencer and MacDougall‐Shackleton 2011; Walker et al. 2013; Kubli and MacDougall‐Shackleton 2014). Signal characteristics are also likely to be influenced by aspects of the signaller's current state (Thomas 1999; Thomas and Cuthill 2002), yet our understanding of the impacts of current state on acoustic signaling remains relatively poorly developed. Acoustic signal performance (e.g., production rate or signal duration) is especially likely to be impacted by current state due to the immediate resource demands associated with producing acoustic signals (Thomas 1999; Barnett and Briskie 2007; Gillooly and Ophir 2010; Zollinger and Brumm 2015). As such, male signal performance may be strongly tied to current state via resource‐allocation trade‐offs with other traits (Sheldon and Verhulst 1996; Olson and Owens 1998; Von Schantz et al. 1999).

Understanding how and why current state impacts signal performance is important because state‐dependence may impact communication systems in a number of ways. First, individuals that are better at locating and defending resources or resisting disease may tend to be in superior states, providing the potential for state‐dependent signals to advertise these aspects of quality (Gil and Gahr 2002; Gillooly and Ophir 2010). Second, if variation in current state generates within‐individual variation in signal characteristics, then this may reduce the utility of those characteristics for assessing between‐individual differences that arise from genetic or early‐life effects (Spencer and MacDougall‐Shackleton 2011; MacDougall‐Shackleton and Spencer 2012). Finally, where receivers can monitor within‐individual changes in signal performance by senders over time, negative impacts of current state may permit receivers to identify individuals in weakened states, which might then be profitably targeted (e.g., in intrasexual competition) or actively avoided (e.g., in mate choice).

A number of studies conducted in non‐social species have investigated how male song production relates to, or is affected by the activation of, the immune response (Duffy and Ball 2002; Garamszegi 2004; Owen‐Ashley et al. 2006; Dreiss et al. 2008; Munoz et al. 2010; Kubli and MacDougall‐Shackleton 2014). In each case, some form of resource‐allocation trade‐off between song production and immunity has been implicated, and in three cases there is experimental evidence that activation of the immune response reduced song performance (Garamszegi 2004; Dreiss et al. 2008; Munoz et al. 2010). However, whether the song performance of dominant males in cooperatively breeding societies is sensitive to variation in current state, and more specifically the mounting of immune responses, is not yet known (Double and Cockburn 2003; Seddon et al. 2004). In cooperatively breeding societies, reproductive skew is typically marked and both within‐ and extra‐group challengers may benefit from identifying dominant individuals in transiently weakened states, in order to contest their dominant breeding position.

Here, we use experimental immune‐system activation to test whether the song performance of dominant male white‐browed sparrow weavers (*Plocepasser mahali*) is affected by current immune state. White‐browed sparrow weavers are cooperatively breeding birds that hold year‐round territories and live in groups comprising a dominant pair (who monopolise within‐group reproduction) and up to 10 non‐breeding subordinates of both sexes that can be of natal or immigrant origin (Harrison et al. 2013a). Approximately 15% of offspring are sired by extra‐group males (Harrison et al. 2013a), and females are more likely to cuckold social partners of low heterozygosity (Harrison et al. 2013b), suggesting that indicators of male health could indeed play a role in mate choice and/or intrasexual competition in this species. Dominant males produce a breeding season song almost exclusively during the dawn chorus (York 2012; York et al. 2014).

Specifically, we investigate whether the song performance of dominant males differs following exposure to phytohemagglutinin (PHA) compared to controls (exposed to phosphate buffered saline [PBS]). PHA activates the immune system, principally the cell‐mediated immune response, which carries appreciable energetic costs (Martin et al. 2003, 2006). PHA challenge was utilized for immune stimulation given the ease of confirming successful immune activation in the field, and because the associated cell proliferation (which draws on energetic and antioxidant reserves) would begin to occur before the quantification of song performance at dawn on the following day (Martin et al. 2003, 2006; Cram et al. 2015). We predict that if dawn song performance is negatively impacted by immune activation, PHA‐challenged males should exhibit greater within‐individual reductions in song performance than controls. Because the extent of local swelling response to PHA injection reflects the infiltration of immune cells into the local area (Martin et al. 2006; Salaberria et al. 2013), we also investigate the possibility that a male's song performance prior to PHA challenge (a potential indicator of his quality) may signal his capacity to mount an immune response to that challenge. We predict that pre‐challenge song performance will positively predict the swelling response in PHA‐treated males, as those males able to invest more in song performance would also have a greater capacity to allocate resources to mounting a swelling response (Reznick et al. 2000).

## Materials and Methods

**Figure 1:**
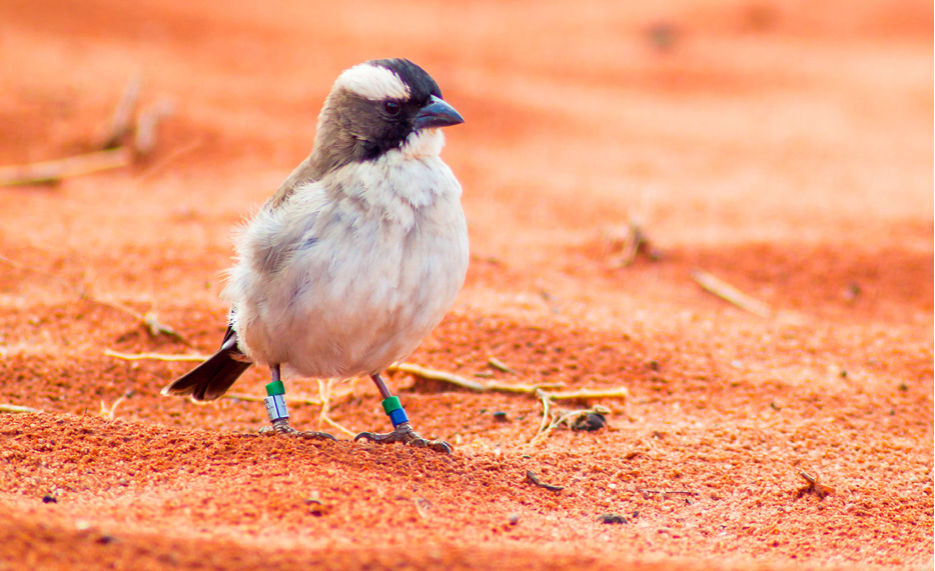
A male white‐browed sparrow weaver, *Plocepasser mahali*, in Tswalu Kalahari Reserve, South Africa. Photo by Jenny York.

Data were collected between November 2010 and March 2011 on a color‐ringed population (SAFRING license 1444) of white‐browed sparrow weavers (a male is shown in Fig. 1) that have been monitored intensively since 2007 (Harrison et al. 2013a,b; York et al. 2014), at the Tswalu Kalahari Reserve, South Africa (27°15′S, 22°26′E). Specific individuals were captured in a targeted manner by flushing them from their individual over‐night roost chamber into a purpose‐built capture net (Wingfield and Lewis 1993; Cram et al. 2014).

To standardize for breeding status, as the study was conducted during the breeding season, only dominant males that did not have an ongoing breeding attempt on their territory at the time of the experiment (no eggs or nestlings present) were used. Dominant males were assigned to two treatment groups: 12 to be PHA‐challenged and 12 to be PBS‐controls, with one from each treatment assessed on the same 2 days and treatment order was alternated between experimental pairs. Due to external factors, not all males were sampled successfully for all metrics at all stages of the experiment and so sample sizes vary slightly among analyses. Two males were not successfully caught after song recording on day 1 (both of which had been assigned to the PHA treatment) and so these males have been excluded from all analyses. Four males were not successfully recorded at dawn on day 2 (one PHA, three PBS), and so could not be included in the analysis of the song response to immune challenge. Seven males (three PHA treated, four PBS treated) evaded capture on evening 2, and so could not be included in the analysis of the body mass response to immune challenge. Final sample sizes are provided for each analysis in the Results.

All captures, handling and measurements were conducted by the same person (J. E. Y). On the evening of day 1, body mass was measured to the nearest 0.1 g using an Ohaus CS200 scale (Ohaus Corporation, Ohaus Europe GmbH, Nänikon, Switzerland). The thickness of patagium (wing‐web) of the right wing was measured three times using a pressure sensitive calliper (Mitutoyo 700‐118). Then birds were injected subcutaneously with either a solution of 0.02 mg PHA (L8754 SIGMA, Sigma‐Aldrich Company Ltd., Gillingham, Dorset, United Kingdom) in 0.04 mL autoclaved PBS (P4244 SIGMA), or a control solution of 0.04 mL PBS (following: Spottiswoode 2008; Cram et al. 2015). Following injection, the male was returned to his roost chamber (total handling time of 27.0 ± 0.6 min (mean ± SE) from capture). Males were re‐caught 24 h later (24.2 ± 0.1 h, mean ± SE). On the second capture, three measurements of the right patagium were made, and body mass was recorded. Wing‐web thickness measurements were conducted three times in quick succession (on evening 1 and 2) and these measurements were highly repeatable (*F* = 709.82, *r* = 0.99, *P* < 0.001) (Lessells and Boag 1987). Given the high repeatability, the difference between the pre‐challenge mean and post‐challenge mean patagium thicknesses for each male was calculated, and is hereafter referred to as the swelling response.

The dawn song performance duration of each male was quantified on the consecutive dawns of day 1 and day 2, with the immune challenge carried out on evening 1 and assessment of the local swelling response to the challenge on evening 2. All behavioral and song definitions and methods follow those in York et al. (2014). Song performance start time was when the first syllable of dawn song was produced, and song performance end time was when the last syllable of dawn song was produced; the focal males were watched continuously throughout that period. Dawn song performance duration was thus calculated as the difference between these two time points (song performance start time and song performance end time), to characterize the period of time during which the male was producing song.

Song recordings were made from within 20 m of the male, using a Sennheiser ME66 directional microphone with a K6 power module (2004 Sennheiser UK Ltd, Marlow, Buckinghamshire, United Kingdom), and a Marantz PMD660 solid‐state recorder (D&M Holdings Inc., D&M Audiovisual Ltd., Belfast, Northern Ireland). Song rates were calculated for the 3 min period directly after roost emergence, defined as the moment at which the male left his over‐night roosting chamber based on direct observation. Song rates were sampled at this point to standardize recording quality, before the male began movements around his territory. Song rate was calculated via visual inspection of spectrograms in Avisoft‐SASLab Pro 5.1.16 (R. Specht, Berlin, Germany), following methods in previous studies of this species (York et al. 2014).

All statistical analyses were conducted using R 3.0.1 (R Core Team, 2015). Parametric statistics were used in all cases, having first checked for normality and homoscedasticity. Analysis of covariance (ANCOVA) was used to investigate the effect of treatment on the post‐challenge value of each focal trait, while (1) controlling for among‐male variation in the pre‐challenge value of the focal trait (by fitting this as a covariate predictor), and (2) allowing for the possibility that the magnitude of the treatment effect on the focal trait was dependent upon the males' pre‐challenge trait values (by also fitting the interaction between treatment and pre‐challenge trait value; Crawley 2007). In all cases, the covariate had a significant positive effect on the response term, but did not interact significantly with treatment (see Results). No significant pre‐challenge biases existed between the treatment groups in either song performance duration (Welch two‐sample *t*‐test: *t* = 0.15, *P* = 0.88), song rate (*t* = −0.60, *P* = 0.56) or body mass (*t* = −0.80, *P* = 0.44).

A general linear model (GLM) was used to investigate whether pre‐challenge song performance duration predicted a treatment‐dependent wing‐web swelling response, by testing for an interaction between pre‐challenge song performance duration and treatment. In all analyses, the significance of explanatory variables was calculated on the basis of the change in deviance in the fit of a model when that particular term was removed (Crawley 2007).

## Results

PHA‐challenged males displayed a significantly larger wing‐web swelling response (mean ± SE = 0.40 ± 0.05 mm, *n* = 7) than PBS‐control males (0.07 ± 0.01 mm, *n* = 8), confirming that the immune‐challenge procedure had the expected effect (Welch two‐sample *t*‐test: *t* = 7.08, *P* < 0.001).

**Figure 2:**
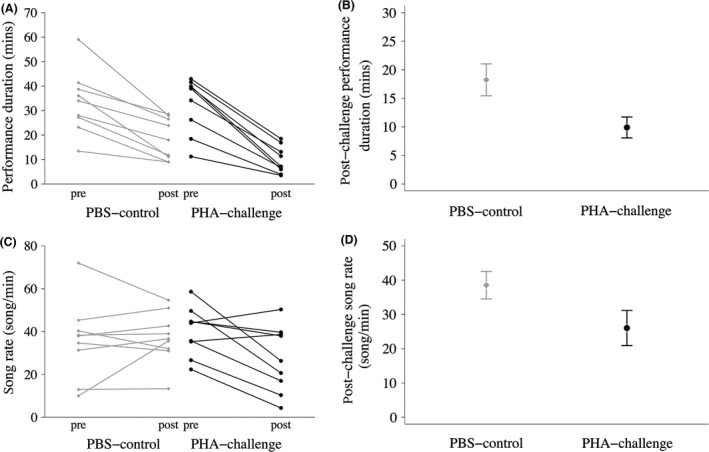
(A) Pre‐ to post‐challenge dawn song performance duration for phytohemagglutinin (PHA)‐challenged (*n* = 9) and phosphate buffered saline (PBS)‐control (*n* = 9) males; (B) mean ± SE post‐challenge dawn song performance duration from model predictions controlling for among‐male variation in pre‐challenge levels; (C) pre‐ to post‐challenge song rate for PHA‐challenged (*n* = 9) and PBS‐control (*n* = 9) males; (D) mean ± SE post‐challenge song rate from model predictions controlling for among‐male variation in pre‐challenge levels.

PHA‐challenged males produced significantly shorter post‐challenge dawn song performances than controls (ANCOVA: *F* = 13.37, *n* = 18, *P* = 0.002; Fig. 2A; while controlling for among‐male variation in pre‐challenge performance duration: *F* = 19.52, *n* = 18, *P* < 0.001), and there was no significant interaction between treatment and pre‐challenge dawn song performance duration (*F* = 0.59, *n* = 18, *P* = 0.45). PHA‐challenged males also exhibited significantly lower post‐challenge song rates than did controls (*F* = 5.54, *n* = 18, *P* = 0.032; Fig. 2B; while controlling for among‐male variation in pre‐challenge song rate: *F* = 9.55, *n* = 18, *P* = 0.007), and the interaction between treatment and pre‐challenge song rate was not significant (*F* = 0.24, *n* = 18, *P* = 0.63).

There was no significant effect of treatment on post‐challenge body mass (ANCOVA: *F* = 1.22, *P* = 0.29; PHA: mean ± SE = 48.7 ± 0.9 g, *n* = 7; PBS mean ± SE = 47.3 ± 0.9 g, *n* = 8; while controlling for variation in pre‐challenge body mass: *F* = 234.01; *n* = 15, *P* < 0.001), and the interaction between treatment and pre‐challenge body mass was not significant (*F* = 0.52, *n* = 15, *P* = 0.48). Furthermore, pre‐challenge body mass was not significantly correlated with pre‐challenge song performance duration (Linear regression: *F* = 1.27, *n* = 18, *P* = 0.27).

**Figure 3:**
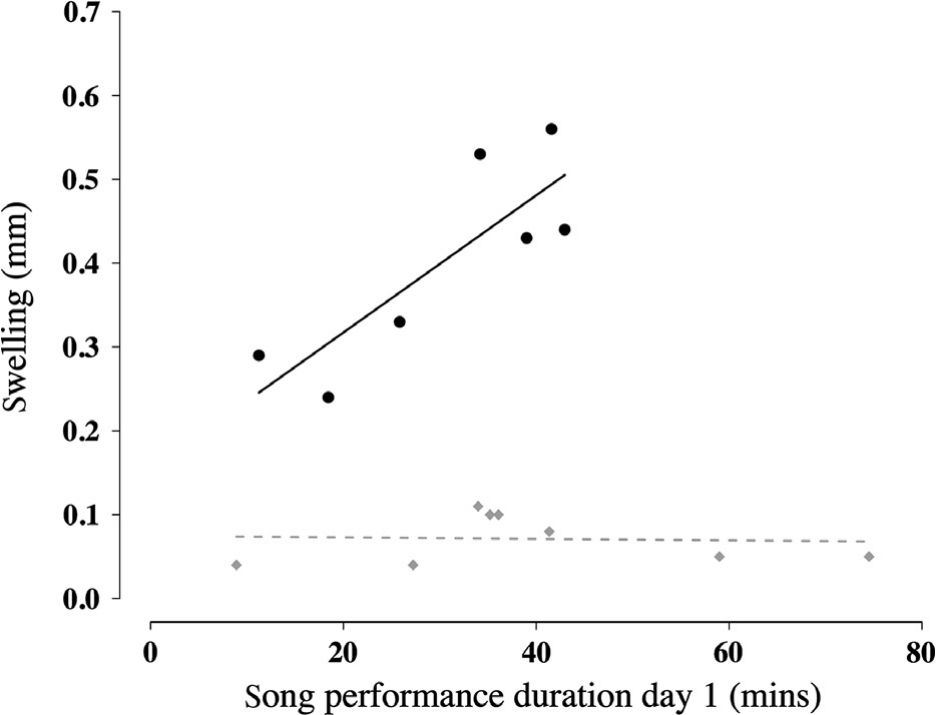
Pre‐challenge dawn song performance duration positively predicts swelling response in phytohemagglutinin males (black circles, *n* = 7) but not in phosphate buffered saline males (gray diamonds, *n* = 8, linear regression lines of best fit).

Finally, a male's pre‐challenge song performance duration predicted his swelling response to the challenge in a treatment‐dependent manner (pre‐challenge song duration × treatment interaction: GLM: *F* = 15.39, *n* = 15, *P* = 0.002; Fig. 3). Pre‐challenge dawn song performance duration positively predicted the extent of the swelling response in PHA‐challenged males (Linear regression: *t* = 3.33, *n* = 7, *P* = 0.021), but did not significantly predict the swelling response of PBS‐injected controls (*t* = 0.14, *n* = 8, *P* = 0.89).

## Discussion

Our study provides experimental evidence that immune‐system activation negatively impacts the song performance of dominant males in a cooperatively breeding bird. PHA‐challenged males exhibited significantly reduced post‐challenge dawn song performance durations and song rates relative to controls. This finding concords with those of previous studies of solitary or pair‐breeding species, in which immune challenges have been found to impact aspects of sexual signaling (Garamszegi 2004; Dreiss et al. 2008; Munoz et al. 2010). Our findings provide the first experimental evidence that the song performance of dominants in a cooperatively breeding society is impacted by their current state, and therefore highlights the potential for within‐ and extra‐group same‐sex competitors to monitor local dominants acoustically with a view to challenging weakened individuals.

In the current study, we have demonstrated that information about dominant males' current state is available in the form of simple performance metrics that receivers could readily assess (song rate and performance duration). While we were unable to quantify song rates throughout the entire post‐emergence period of the song performance, there was a significant effect of the immune challenge on song rate during the period immediately after emerging from the roost (while still in close proximity to other members of the social group in their individual overnight roost chambers). Indeed, it is likely that receivers within the group's roosting trees may necessarily utilize a similar sampling strategy to assess the dominant male, before he begins to move around the territory.

One possibility that we were unable to address in the current study is that immune activation could result in changes to other aspects of the “quality” of the song produced. It would be of value to investigate this because aspects of the quality of song (such as the production of specific acoustic characteristics, song repertoire usage or the consistency of individual syllables produced) may also decline following an immune challenge or, alternatively, may increase in order to mitigate the effects of producing less song in total. However, it is unclear at present which acoustic components of song encode differentially valuable information in white‐browed sparrow weavers. Therefore, future work on this system would first need to investigate which aspects of acoustic song structure are associated with male “quality” and whether these components are used by receivers, in order to ask whether immune challenge affects their production. Nevertheless, it is certainly possible that simple song performance metrics (as quantified in the current study) are sufficient for receivers to assess male current state, given that song performance does indeed vary in response to activation of the immune system.

The most commonly invoked explanation for signaling–immunity interactions is a resource allocation trade‐off (Sheldon and Verhulst 1996). Under this logic, the observed effect of PHA‐challenge could reflect a strategic reduction in song output, to release resources for the immune response and/or mitigate its net impact on resource availability for other essential processes. Indeed, our finding that males with longer pre‐challenge song performances subsequently mounted stronger swelling responses to PHA is consistent with song and immunity drawing on a common pool of resources, whose size may vary among males. While changes in body mass can elicit condition‐dependent changes in song performance (Cuthill and Macdonald 1990; Thomas and Cuthill 2002), the observed impact of PHA on song output cannot be readily attributed to an effect on body mass in the current study, as the body mass measures taken were unaffected by treatment. Instead, PHA‐challenged males may have mitigated impacts of the challenge on their body reserves by reallocating resources from elsewhere via either the down‐regulation of song and/or by elevating resource intake due to activational behavioral plasticity (Snell‐Rood 2013). Alternatively, PHA‐challenged males may have suffered net deficits in key micronutrient resources that are not reflected in body mass (for example, vitamins and antioxidants), given the role that these nutrients can play in sexual signal production (Olson and Owens 1998; Von Schantz et al. 1999). However, it is important to keep in mind that the histological response to PHA is not currently characterized for white‐browed sparrow weavers, which hinders our ability to interpret the specific links between the underlying immunology and the behavioral response to PHA challenge. That said, the results from the use of this nonpathogenic challenge seem likely to provide a conservative estimate of the effects of a real infection on song performance, given the additional costs that would be experienced by an infected individual (e.g., direct costs of parasite proliferation and tissue damage).

A second less‐commonly considered explanation for trade‐offs between immune activation and signaling is that mounting an immune response may increase the risk of predation, for example due to a consequent loss of condition or via impacts of inflammatory responses on aspects of mobility (Owen‐Ashley and Wingfield 2007). Where this is the case, selection may have favored immune responses that also elicit adaptive sickness behaviors, not only to release resources for immunological processes, but also to reduce other risk‐enhancing activities such as the production of conspicuous displays (Owen‐Ashley and Wingfield 2007). A mechanism of this kind could conceivably account for our findings, as dawn song production in particular may leave males vulnerable to predation, as low light levels may inhibit predator detection and preclude an effective escape, while broadcast song can reveal location (Krams 2001; Berg et al. 2006). For diurnal species, both resource constraints and predation risk may be at their most acute at dawn, due to overnight loss of condition and low light levels that may inhibit predator detection and evasion. Dawn song performance in particular might therefore be expected to be sensitive to current state. It would be illuminating to investigate the spatial movements associated with dominant male song performance at dawn. It is possible that a decrease in movements during the dawn song performance may also play a role in risk‐avoidance strategies whilst undergoing an immune response.

By demonstrating that activation of the immune response negatively impacts dawn song performance, our findings highlight the possibility that receivers might be able to identify individuals in weakened states. Receivers could potentially achieve this by monitoring within‐individual changes in song performance characteristics over time, which would be plausible in territorial and social species. This type of information may be of use in both mate choice and intrasexual competition. While the reproductive benefits to females of identifying potential mates of high quality are widely appreciated (Gil and Gahr 2002), attending to performance as an indicator of current state might additionally allow females to minimize their exposure to infectious males (Sheldon 1993; Loehle 1997). Indeed, dominant female white‐browed sparrow weavers may utilize dawn song in this way as they are more likely to mate extra‐group when their social partners are of low heterozygosity (Harrison et al. 2013b). The rejection of social mates in poor current health (a plausible consequence of low heterozygosity at immune‐relevant loci; Drury 2010) provides one potential candidate mechanism.

Male receivers could also benefit substantially from attending to signals that reveal information about current state, as this may facilitate the targeting of temporarily weakened rivals in competition for resources or mates. While in most studies of dawn song, intrasexual competitors reside outside the singer's territory, dominant male white‐browed sparrow weavers may face challenges from their resident subordinates as well as extra‐group males (Lewis 1981, 1982; Harrison et al. 2014). In this species and other group‐living birds, dawn song may therefore function in part to display a male's current state to his own resident subordinates as well as extra‐group competitors. Indeed, when males are year‐round resident on stable, densely packed territories (as is the case in this species; Lewis 1982; Harrison et al. 2013a,b), local receivers might be readily able to undertake the repeated within‐individual sampling of song characteristics over time that may be needed to identify transient or progressive weaknesses in the state of individual singers. Even where sequential assessment of individuals is impossible, however, receivers may still be capable of gleaning information about the current state of signalers from inter‐individual comparisons alone, where inter‐individual variation in performance characteristics arises in large part from variation in current state. Indeed, the dawn chorus in particular may provide an ideal forum for this, by facilitating comparisons of the performances of multiple males under identical environmental conditions. Future studies might now profitably investigate the extent to which subordinates in social species do indeed utilize the song performance of dominants when making adaptive decisions about whether and when to challenge for the dominant position.

## Conflict of Interest

None declared.
